# Supplementary figures and images for: A Standardized Nonvisual Behavioral Event Is Broadcasted Homogeneously across Cortical Visual Areas without Modulating Visual Responses
Source: eNeuro. 2022 Sep 21;9(5):ENEURO.0491-21.2022. doi: 10.1523/ENEURO.0491-21.2022 (PMC9512619; doi:10.1523/ENEURO.0491-21.2022)

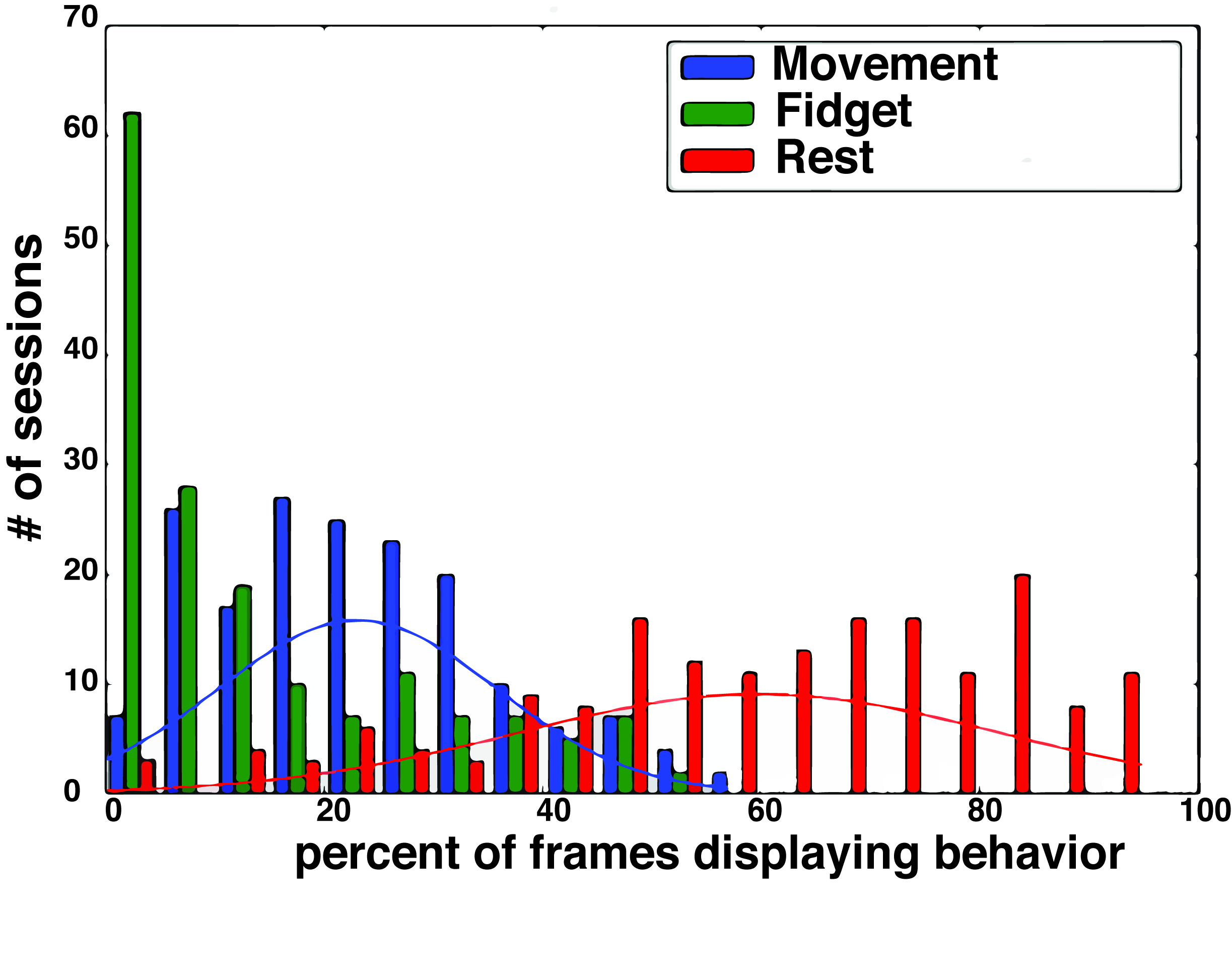

Supplement: Extended Data Figure 2-1 — Fidget rate frequency across sessions. a, Left, Bar plot visualizing the number of sessions (total of 144) with their percentage of video frames classified as one of three behaviors (movement, resting, or fidget). Download Figure 2-1, TIF file. [file enu-eN-NWR-0491-21-s02.tif]

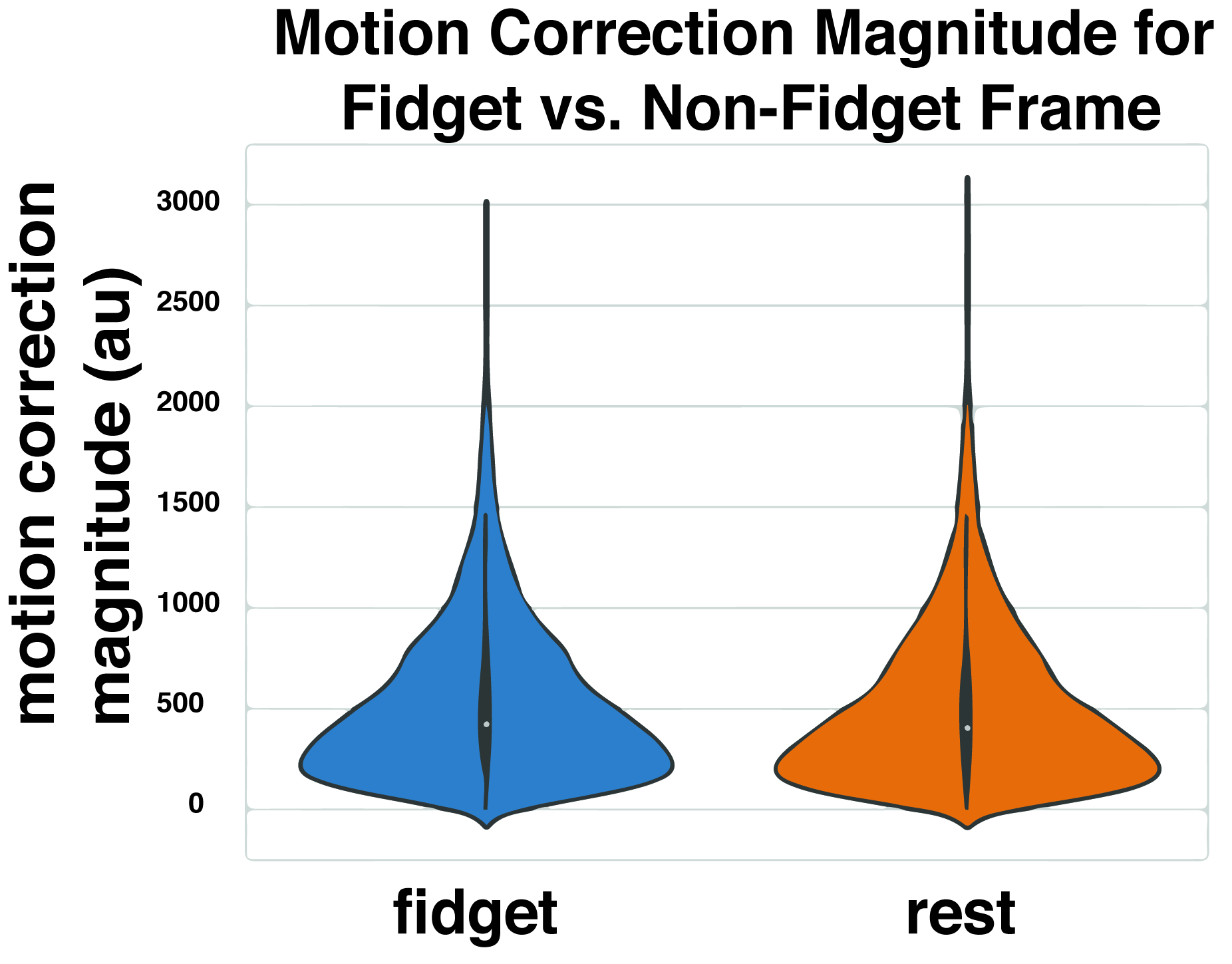

Supplement: Extended Data Figure 3-1 — Fidgets do not induce translational movement of the cortex. Violin plots of the Euclidean distance of 2P image motion in the x and y dimensions (translational movements) during mouse fidget versus mouse resting state (no movement). Download Figure 3-1, TIF file. [file enu-eN-NWR-0491-21-s04.tif]

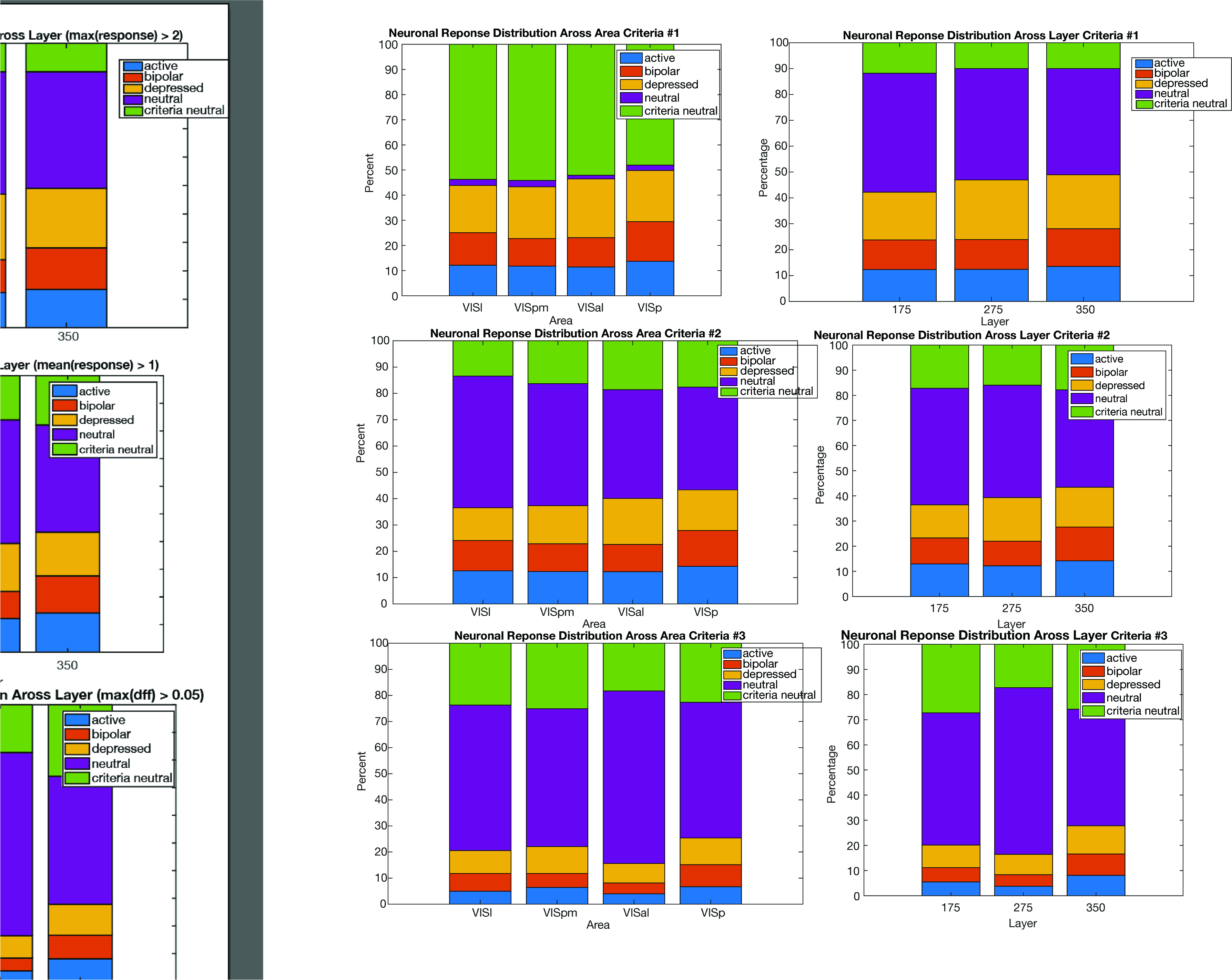

Supplement: Extended Data Figure 3-2 — Equal distribution of clustered neuronal response types across area and layer is robust to threshold criteria. Distributions of clustered neuronal responses after applying three different threshold criteria to neural data. The distributions are conditioned in area or layer, and neural responses that did not meet the threshold criteria are labeled in green as criteria neutral. Depending on the strictness of the criteria the percent of neutral neurons changes, but the distribution of neural response types conditioned on area or layer remains fairly consistent. Download Figure 3-2, TIF file. [file enu-eN-NWR-0491-21-s05.tif]

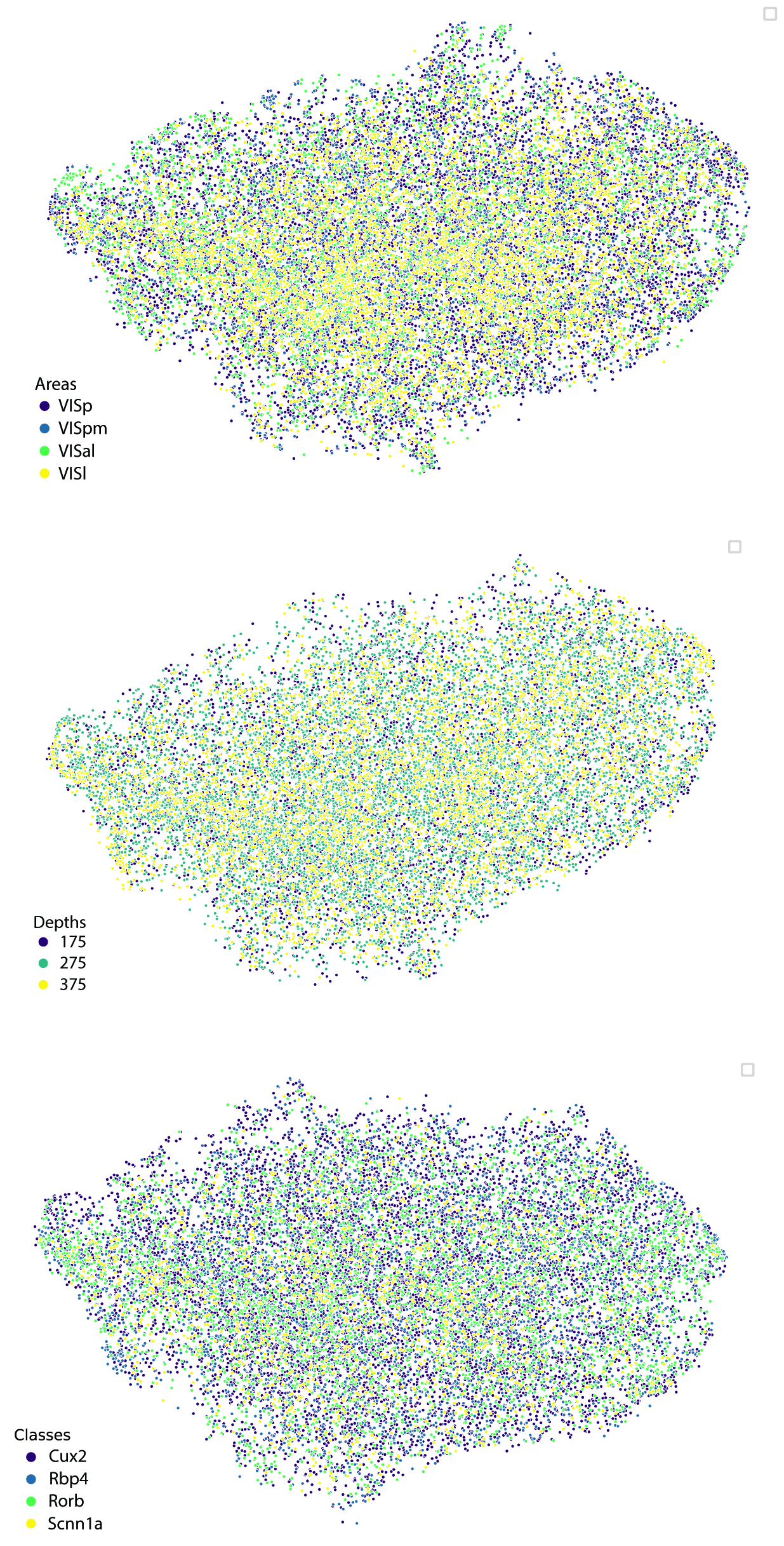

Supplement: Extended Data Figure 3-3 — UMAP embeddings of postfidget single neuron responses by area, layer, and mouse Cre-line. Visualization of the projection of postfidget neural responses from averaged single neurons into a 2D-embedded space identified by unsupervised nonlinear dimensionality reduction (UMAP), labeled by area (top subplot), layer (middle subplot), and mouse Cre-line (bottom subplot). Download Figure 3-3, TIF file. [file enu-eN-NWR-0491-21-s03.tif]

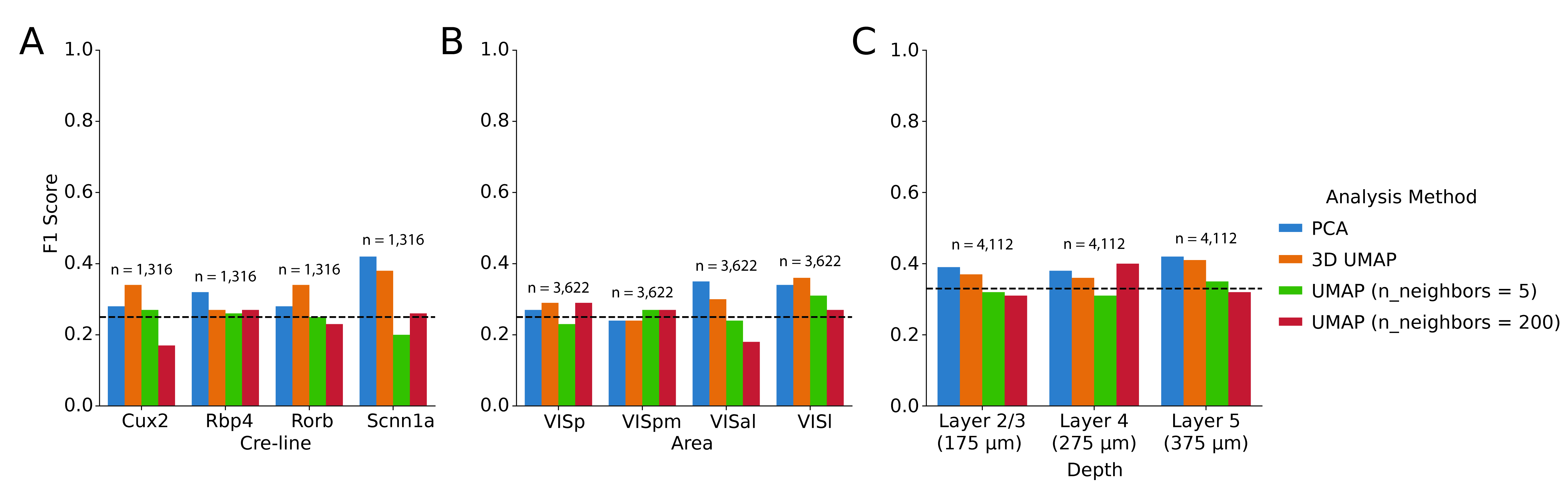

Supplement: Extended Data Figure 3-4 — Lack of neuronal activity differences between Cre-line, area, and depth is not due to the method of dimensionality reduction or hyperparameter choice. A gradient boosted decision tree classifier with five-fold cross-validation (as in Fig. 3e,f) was trained to differentiate cells according to Cre-line, visual area, and laminar depth based on their dimensionality reduced fidget-onset aligned neuronal calcium imaging traces. The dimensionality reduced traces were compared using four methods: PCA of the first 10 principal components, UMAP in the first three dimensions (3D UMAP), UMAP with n_neighbors = 5, and UMAP with n_neighbors = 200. Chance performance is shown as a dashed horizontal line and was obtained by training a classifier on randomly permuted labels. a, F1 scores for a single classifier trained to distinguish whether neuronal activity of a single cell from one of four Cre-lines: Cux2, Rbp4, Rorb, and Scnn1a. b, The same procedure as in a but for another single classifier trained to distinguish single neurons by the visual area (VISp, VISpm, VISal, and VISl) they were collected from. c, The same procedure as in a, b but for a classifier trained on the laminar depth of a neuron. Download Figure 3-4, TIF file. [file enu-eN-NWR-0491-21-s06.tif]
